# Translating Evidence from Clonal Hematopoiesis to Cardiovascular Disease: A Systematic Review

**DOI:** 10.3390/jcm9082480

**Published:** 2020-08-02

**Authors:** Veronica Papa, Luisa Marracino, Francesca Fortini, Paola Rizzo, Gianluca Campo, Mauro Vaccarezza, Francesco Vieceli Dalla Sega

**Affiliations:** 1 Department of Motor Sciences and Wellness (DiSMeB), Università Degli Studi di Napoli “Parthenope,” 80133 Napoli, Italy; veronica.papa@uniparthenope.it; 2 FAPAB Research Center, 96012 Avola (SR), Italy; 3 Department of Morphology, Surgery and Experimental Medicine and Laboratory for Technologies of Advanced Therapies (LTTA), University of Ferrara, Via Fossato di Mortara 64/B, 44121 Ferrara, Italy; luisa.marracino@student.unife.it; 4 Translational Research Center, Maria Cecilia Hospital, GVM Care & Research, Cotignola, 48033 Ravenna, Italy; frtfnc@unife.it (F.F.); cmpglc@unife.it (G.C.); vclfnc@unife.it (F.V.D.S.); 5 Department of Medical Sciences, Cardiovascular Institute, Azienda Ospedaliero-Universitaria of Ferrara, University of Ferrara, 44124 Cona, Italy; 6 School of Pharmacy and Biomedical Sciences, Faculty of Health Sciences, Curtin University, Curtin Perth Campus, Bentley, Perth, WA 6102, Australia

**Keywords:** clonal hematopoiesis of indeterminate potential, driver mutation, DNMT3A, TET2, atherosclerosis, coronary heart disease

## Abstract

Some random mutations can confer a selective advantage to a hematopoietic stem cell. As a result, mutated hematopoietic stem cells can give rise to a significant proportion of mutated clones of blood cells. This event is known as “clonal hematopoiesis.” Clonal hematopoiesis is closely associated with age, and carriers show an increased risk of developing blood cancers. Clonal hematopoiesis of indeterminate potential is defined by the presence of clones carrying a mutation associated with a blood neoplasm without obvious hematological malignancies. Unexpectedly, in recent years, it has emerged that clonal hematopoiesis of indeterminate potential carriers also have an increased risk of developing cardiovascular disease. Mechanisms linking clonal hematopoiesis of indeterminate potential to cardiovascular disease are only partially known. Findings in animal models indicate that clonal hematopoiesis of indeterminate potential-related mutations amplify inflammatory responses. Consistently, clinical studies have revealed that clonal hematopoiesis of indeterminate potential carriers display increased levels of inflammatory markers. In this review, we describe progress in our understanding of clonal hematopoiesis in the context of cancer, and we discuss the most recent findings linking clonal hematopoiesis of indeterminate potential and cardiovascular diseases.

## 1. Introduction

A growing body of evidence is pointing at clonal hematopoiesis of indeterminate potential (CHIP), defined as “the presence of clones in the absence of overt oncologic disease”, as a novel risk factor linked to the development of hematological cancers or cardiovascular diseases later in life (for a comprehensive view on the field, see [1]). 

Our review discusses the definitions and significance of clonal hematopoiesis (CH) and the specific characteristics of CHIP, helps orientate the non-oncology expert to the jargon of this new interdisciplinary field and aims to underscore the links between CHIP, malignancy risk and the surprising risk in developing cardiovascular disease (CVD). Furthermore, based on the most recent knowledge in the field, it discusses a few potential methods to combat and eventually mitigate the pathogenic potential of mutated clones. 

To fulfill the aims of our manuscript, we performed a systematic review following the preferred reporting items for systematic reviews and meta-analyses (PRISMA) amendment to the quality of reporting of meta-analyses (QUOROM) statement. The search strategy was performed between October 2019 and May 2020. The terms searched were (clonal hematopoiesis) AND (myocardial OR cardiovascular OR valvular OR thrombus OR heart failure OR infarction OR atherosclerosis OR coronary). Only articles published in English and peer-reviewed journals were selected. The search was carried out on PubMed, Biomed Central, Google Scholar, and Cochrane Library. The inclusion criteria were observational studies focused on CHIP and its association with any type of cardiovascular disease. We gave preferences to citations within the past five years; studies published before the first study about CHIP were excluded. A total of 135 records were screened. For all articles, abstracts were reviewed, and the full reference list was analyzed. All the authors agreed on the final number of studies included. 

## 2. Age-Related Clonal Hematopoiesis and Cancer 

The first association between the presence of clonal expansion and cancer dates back to 1976 when Peter Nowell proposed that cancer could arise from a single cell, and stated that tumor progression might result from acquired genetic variability within the original clone, allowing sequential selection of more aggressive sub-lines [2,3]. Consistent with that, Stratton and collaborators, in 2009, defined cancer development as analogous to the Darwinian evolution process: both of them are characterized by the continuous acquisition of heritable genetic variation and driven by natural selection acting on the resultant phenotypic diversity [4]. Moreover, according to the definition by Cooper and collaborators [5], clones are identical cells within a cellular population; consequently, clonality refers to a uniform population of cells that could be either wild-type or mutant and eventually malignant. The concept of clonality, although historically referring to myeloid and lymphoid neoplasms, could also be applied to normal hematopoiesis. In this regard, clonality in normal hematopoiesis has been elegantly demonstrated [6], also highlighting the fact that hematopoietic stem/progenitor cells’ (HSPC) clonality can be restored and reactivated [6].

Most somatic mutations occurring in the DNA sequence of a cell allow limited abnormal growth potential and have no effects on cell proliferation, or cause limited benign growth; occasionally, a single cell acquires a set of sufficiently advantageous mutations that allow it to proliferate autonomously, invade tissues and metastasize. Somatic mutations may be defined either as “driver,” i.e., those who contribute to clonal expansion, or “passenger,” i.e., those who do not. However, either mutation could indicate the presence of a clone; although driver mutations lead to clonal expansion, they may not necessarily be associated with malignant neoplasia. It might, therefore, be assumed that every somatic mutation occurring in cancer genes, able to give a growth advantage to the cell carrying the mutation, represents a driver mutation (for further details, see Table 1).

It has been estimated that epithelial cancers, such as breast, colorectal, or prostate cancer, require several driver mutations (see Table 1), whereas cancers of the hematological system may need fewer [7]. Well-defined cancers’ precursor states, such as adenomatous colon polyps or dysplasia of the uterus or cervix, have illuminated the multistep process of oncogenesis. Hematological malignancies’ precursor states, in contrast, do not have any anatomical correlates: blood cell counts and bone marrow morphology in a hematological precursor state may be, in fact, entirely normal; these states being instead defined by markers of clonality like the recently described clonal hematopoiesis [8,9,10]. 

Blood stem cells acquire random somatic mutations with each cell division over time. Most of these mutations have little or no effect on normal cell physiology, but eventually, some of them could provide a selective advantage to the hematopoietic cell in which they occur. The result is a clonal expansion in the peripheral blood since the mutated stem cells maintain the ability to differentiate into granulocytes, monocytes, and lymphocytes [11,12,13,14,15,16,17]. 

CHIP has been recently defined by Steensma as a process in which somatic mutations in leukemia-associated driver genes result in the expansion of a clone of bone marrow and peripheral blood cells [9]; it is, therefore, a clonal expansion of hematopoietic cells with a somatic mutation in the presence of otherwise normal white blood cells [11]. More specifically, CHIP refers to a CH with a variant allele fraction of at least 2% or higher in blood or bone marrow samples without a diagnosis of other hematological conditions, thus distinguishing this particular condition from pre-leukemic states such as myelodysplastic syndrome (MDS). Other main features of CHIP include the absence of morphological variation in the blood cells as well as diagnostic criteria for symptoms currently associated with hematological cancer (for example, gammopathy) [13,14]. The prevalence of CHIP varies with age, ranging from less than 1% in those younger than 40 years, to more than 15% in those 90 years and older [12,18,19]. Generally, the clonal mosaicism for a large chromosomal abnormality, reflecting the expansion of a specific cell clone, appears to arise in approximately 2% of the elderly population [20]. Criteria for CHIP definition are summarized in Table 2.

The most commonly mutated genes in CHIP are the DNA methyltransferase 3A (DNMT3A), the DNA demethylase TET2, and the transcription regulator ASXL1. Mutations in these three genes are commonly associated with MDS and acute myeloid leukemia (AML), providing a selective advantage to the hematopoietic stem cell in which they occur [18,19,20,21,22]. TET2 mutations have also been described as leukemia-associated events that appear to be present in up to 25% of patients with myeloid neoplasm [23]. Moreover, DNA methylation patterns change with age, and alterations in the DNA methylation process have recently been associated with atherosclerosis as well as with MDS and its drug responsiveness [24,25]. Methylation of the promoter region is frequently used by the cell to inactivate gene expression, and DNA methyltransferase inhibitors can induce the re-expression of previously methylated silenced gene products [26]. Mutations in the ASXL1 gene are associated with MDS and chronic myelomonocytic leukemia [27,28,29]. More recently, Shlush and collaborators reported that DNMT3A mutations are common in pre-leukemic stem cells [30]. In 2014, three independent groups reported analysis of DNA exome datasets and documented age-dependent mutations in genes associated with hematological cancers. Out of more than 70 different genes being identified, the most frequently mutated were DNMT3A, TET2, ASXL1, TP53, JAK2, SF3B1, CBL, SRSF2, PPM1D, and BCOR [3,19,31]. These mutations were common, showing, for example, an increase in red cell width (which could be a marker of perturbation of hematopoiesis due to the expanded clone) [31]. Genovese and collaborators analyzed whole-exome sequences of the DNA in peripheral blood cells analyzed whole-exome for cancer or other hematological phenotypes. The vast majority of mutations were found in four genes, DNMT3A, TET2, ASXL1 and PPM1D. All of these mutations also occurred with a low allelic fraction, therefore confirming that they were somatic mutations. Of note, CHIP with somatic mutations was detected in 10% of the elderly study participants, with increasing frequency with age; in the tested cohort, up to 7 years after DNA sampling, approximately 42% of subjects who displayed clonality at the time of sampling developed hematological cancer. Importantly, only 10% of the studied population displayed CHIP, and the absolute risk of conversion from clonal hematopoiesis to hematological cancer was found to be as low as 1.0% per year.

Therefore, this study suggests that, although the presence of clones highly correlates with, and is a strong risk factor for these cancers, whole-genome sequencing of blood samples, for screening purposes, would represent a premature and useless approach. 

In the next paragraphs, we will present emerging evidence from epidemiologic and clinical studies of an association between CHIP and cardiovascular disease (CVD). This finding is not surprising since there is compelling evidence showing that cancer and CVD share biological mechanisms underlying their pathogenesis [32]. Inflammation participates in the pathogenesis of both cancer and CVD, and it is involved in the initiation, the progression, and the complication of both malignant tumors and atherosclerotic plaques [33]. Regulation of the inflammatory signaling pathways has resulted in an innovative strategy in the treatment of cancers, as well as in a reduction in the risk of cardiovascular events. Furthermore, the incidences of both cancer and CVDs increase with advancing age [34,35]. Cardiovascular disease is the leading cause of death in the elderly, but almost 60% of elderly patients with atherosclerotic CVD have either no conventional risk factor or just one risk factor [36,37,38].

## 3. CHIP Is Associated with Cardiovascular Diseases 

Jaiswal et al. [31] were the first to report that the presence of CHIP-linked mutations leads to a 40% increase in all-cause mortality, which is, in turn, linked to an increased risk of coronary heart disease and ischemic stroke. They analyzed the whole-exome sequencing data from the peripheral blood DNA of 17,182 subjects, unselected for hematological cancers, in 22 cohorts of three different consortia. Their study confirmed that the most frequent mutated genes in CHIP are DNMT3A, TET2, and ASXL1, and revealed an increased risk of mortality from cardiovascular disease (40%) in comparison to the risk of dying from hematological cancers (4%) in patients carrying a mutation in the CHIP genes. Moreover, the comparison between subjects carrying one or more CHIP mutations and non-carriers showed no significant differences in hematological values except for red cell distribution width. To study more in-depth the relationships between CVD and CHIP, in a later study, Jaiswal et al. carried out whole-exome sequencing on blood samples obtained from four case-control studies consisting of 4726 individuals with coronary heart disease and 3529 control individuals (matched on the basis of age, sex, type 2 diabetes status, smoking history in the prospective cohorts; for more details, see [14]).

Interestingly, CHIP carriers had a risk of coronary heart disease that was 1.9 times higher than non-carriers, which was comparable with what was found in the prior study. In two retrospective case-control studies for the evaluation of early-onset myocardial infarction, subjects with CHIP had a risk of myocardial infarction that was 4.0 times higher in comparison to non-carriers. In these cohorts, mutations in DNMT3A, TET2, ASXL1, and JAK2 were each individually linked to coronary heart disease [14]. These data demonstrate not only that CHIP is an age-related premalignant condition, but that it is also associated with an overall increased mortality rate as well as an increased risk of cardio-metabolic diseases. Importantly, coronary events increased with clone size, and there was also a dose–response relationship between clone size and atherosclerosis based on artery calcification imaging [14]. In this regard, CHIP subjects without coronary heart disease, but carrying a variant allele fraction of a least 10%, had 12 times the risk of having a high coronary-artery calcification score as did non-carriers, whereas participants with a variant allele fraction of less than 10% had no increased risk [14]. 

Dorsheimer et al. [22] further extended these observations: they tested for the presence of CHIP mutations in archived bone marrow DNA samples from 200 patients with chronic heart failure (HF) caused by ischemic pathology, enrolled in different clinical trials evaluating autologous bone marrow treatment. This undertaking yielded several significant findings: firstly, in this population of survivors of acute myocardial infarction, the prevalence of CHIP mutations increased with age, in agreement with the study by Jaiswal et al. [14], since the somatic mutations most frequently occurred in DNMT3A and TET2 genes, and were detected both in bone marrow and peripheral blood samples [22]. Secondly, carriers of CHIP had a marked increase in the risk of death from HF [22], also in accord with prior studies [31]. Kaplan–Meier survival plots, related to patients carrying either DNMT3A or TET2 mutations, clearly documented a worse long-term clinical outcome, both in terms of re-hospitalization due to ischemic events and death [22]. Two commentaries have highlighted the novelty and the importance of Dorsheimer’s study [39,40], since most of the fatalities among patients with CHIP resulted, in fact, from worsening HF and not atherothrombotic events [22].

Moreover, in this group, CHIP carriers also had an increase in the number of hospitalizations for HF [22]. These observations, therefore, support the involvement of CHIP, not only in the pathogenesis of myocardial infarction and stroke, as previously shown [31,39,41], but also in HF. The resulting association of mutant clone size and risk of death in this population could not be explained by CHIP carriers holding a worse HF baseline risk, as measured by alterations in left ventricular ejection fraction, known HF risk scores and serum levels of N-terminal pro-B type natriuretic peptide; thus, HF behaves definitely as a “bona fide” cardiovascular complication of CHIP. 

Recently, Mas-Peiro et al. [40] investigated the effect of the presence of CHIP on the clinical course of aortic stenosis. The study enrolled 279 aortic stenosis patients of which 93 (33%) had mutations in DNMT3A or TET2, the two most common CHIP-driver genes. Ageing renders aortic valve cusps impaired in its functions, with different degrees of sclerosis which can further progress to calcific aortic valve stenosis [42]. In the patients studied by Mas-Peiro et al., CHIP mutations were associated with worse outcomes, including increased mortality after valve replacement during a median 8-month follow-up. The patients with DNMT3A or TET2 mutations displayed a three-fold increase in mortality compared with non-CHIP carriers. Interestingly, CHIP mutations in aortic stenosis patients were not associated with an increase in pro-inflammatory circulating cytokines, but were linked to an inflammatory phenotype in specific immune cell subsets. Specifically, DNMT3A mutation carriers displayed an increase in the Th17 to regulatory T-cell (Tregs) ratio, while TET2 mutations were associated with an increase in the non-classical monocytes. In addition, the same group performed a single-cell RNA sequencing analysis which revealed that the circulating monocytes of patients with aortic stenosis with CHIP driven by DNMT3A or TET2, or HF patients carrying DNMT3A mutations, have an increased expression of IL-1β IL-6 receptor, NLRP3 and CD163, a receptor involved in macrophage activation syndrome [43]. A list of all epidemiological and clinical studies on CHIP and CVD is provided in Table 3.

## 4. Mechanisms by which CHIP Increases Cardiovascular Risk

Epidemiological and clinical studies that have identified the association between CHIP and CVD do not provide information on whether a causal link exists between CHIP and CVD. Similarly, these studies did not explore potential mechanisms underlying the increased risk of CVD in CHIP carriers. For this reason, animal models have been used to investigate whether, and through which mechanisms, CHIP is causally linked to CVD (Table 3). 

These experimental results are discussed in the next paragraphs. Table 3 is a synopsis of the animal and human studies that support the hypothesis of CHIP–driven increase in CVD risk.

### 4.1. TET2 and DNMT3A 

Experiments in genetically engineered mice have shown that CHIP contributes actively to cardiovascular complications. In 2017, Fuster and collaborators focused on TET2, the first gene shown to exhibit a somatic mutation in CHIP [44], known for its involvement in hematopoietic stem cell renewal and cancer [45,46], but so far not associated with CVD development. Mice that lack *Tet2* have accelerated atherosclerosis and a heightened tendency to develop left ventricular dysfunction [44]. Authors observed a clonal expansion into all blood cell lineages, and, regardless the type of diet, a profound effect on diet-induced atherosclerosis in *Tet2* KO mice that displayed plaques in the aortic root which were 60% larger than those in wild-type controls. This finding was paralleled with an increase in the number of macrophages in the intima of the vascular wall, thus suggesting that *Tet2*-deficient hematopoietic cells accelerate atherosclerosis by means of macrophages with enhanced atherogenic activity, probably due to an Interleukin-1ß (IL-1ß)-mediated mechanism. Consistent with the study of Fuster and collaborators [44], Jaiswal and collaborators [14] showed that mice with either heterozygous or homozygous loss of *Tet2* developed aortic root lesions that were 50–70% larger than those in control mice at early time points, while the involved area was also approximately two-fold larger in the descending aorta at later time points. 

One year later, Sano et al. [47] studied the effect of mutations in TET2 or DNMT3A in HF, and reported that, in two experimental mouse models of HF, loss of *Tet2* in bone marrow cells led to reduced left ventricle ejection fraction and increased cardiac fibrosis and remodeling. The same group, in 2018 [48], found that deletion of either *Tet2* or *Dnmt3A* in bone marrow caused increased cardiac hypertrophy and fibrosis and a reduction in cardiac function in mice with angiotensin-II-induced HF, suggesting that the reduced survival rate seen in patients with TET2 or DNMT3A mutations is causally related to altered immune cell function in the myocardium [39,40]. Recently, Sano et al. confirmed the casual relationship between *Tet2*-mutated clone expansion and the development of HF in non-irradiated mice that have not been preconditioned by a myeloablative strategy [49]. In this study, mice receiving *Tet2*-deficient bone marrow cells spontaneously developed age-related cardiac hypertrophy and fibrosis that were associated with an increased inflammatory signature in donor-derived *Tet2*-deficient interstitial macrophages [49]. This observation could be explained by the fact that, in murine macrophages and dendritic cells, *Tet2* represses the transcription of pro-inflammatory molecules such as IL-6, a known pro-atherogenic mediator (even if this IL-6-driven atherogenic effect has been reported to be independent, at least in part, of the well-known enzymatic role of TET2 [39,40]). 

Although findings from clinical studies are in agreement with the mechanistic data obtained in animal models, some evidence seems to contrast with a pro-atherosclerotic role of TET2. Kaasinen et al. [50] recently investigated the effect of TET2 germline mutations in six carriers, representative of three different inactivating mutations, without observing clinical evidence of increased atherosclerosis in those carriers. Macrophages of subjects carrying germinal TET2 mutations did not display increased secretion of IL-1β or IL-8 (50). These findings, in contrast to previous studies, may indicate that TET2 loss may be of limited importance with regard to atherosclerosis. However, it must be considered that germinal TET2 loss, compared to somatic mutations involving only a relatively small fraction of cells, has a wider impact and it is likely to result in the alteration of several processes which could possibly compensate for pro-atherogenic effects. For instance, Kaasinen and collaborators observed an increased expression of chemokine receptor type 4 (CXCR4) in individuals with germinal TET2 mutation that, since CXCR4 exerts an anti-atherosclerotic effect by preserving vascular function in arteries [50], may counterbalance the pro-atherosclerotic effect. In a recent paper, Doring and collaborators [51] demonstrated that CXCR4, the receptor of CXCL12 (whose expression and plasma levels were previously associated with coronary artery disease [52,53]), was able to confer cell-specific athero-protective effects preserving endothelial function in mice.

In contrast, reduced CXCR4 expression was associated with CAD risk in humans, indicating the complex effects of this ligand-receptor axis in atherosclerosis and CAD. Importantly, TET2 loss causes the deregulation of expression of several genes involved in stem cell renewal or differentiation and cancer [54], and can also promote the accumulation of other mutations [55]. Among the genes regulated by TET2 [56] and frequently co-mutated in *Tet2* KO stem cells, there is NOTCH1 [56], a gene known to play a significant role in modulating immune responses and inflammation [57], two factors tightly linked to CVD [58,59]. 

### 4.2. JAK2 Mutations Promote Thrombotic Diseases and Increase Inflammation

JAK2 is a non-receptor tyrosine kinase associated with several cytokine receptors and is critically involved in different processes such as erythropoiesis, atherosclerosis, endothelial cell activation, and myocardium inflammation [60,61,62,63,64]. The JAK2V617F (JAK2^VF^) mutation is linked to myeloproliferative neoplasms (MPN), and in these diseases, it is associated with thromboembolic complications, increased blood viscosity and platelet adhesion, as well as reduced venous blood return [65]. In addition, Wolach et al. have shown that in MPN, JAK2^VF^ promotes acute coronary events and thrombosis by accelerating the formation of neutrophil extracellular traps (NET), components of innate immunity [66]. Importantly, in this study, JAK2^VF^ mutation was also associated with thrombosis in subjects without MPN or other hematologic disorders [66]. Furthermore, Wang and collaborators have shown that hematopoietic JAK2^VF^ expression is associated with increased atherosclerosis, both in its early and advanced stages, as it promotes neutrophil infiltration and plaque instability in a mouse model of hypercholesterolemia [67]. In this model, JAK2^VF^ mutation has also been associated with defects in the functions of erythrocytes and macrophages, which cause increased erythrophagocytosis and abnormal efferocytosis [67].

The role of JAK2 mutations in the context of CHIP has been explored by Sano and collaborators, who found that in a murine model of HF, which shows no sign of MPN phenotype, myeloid-directed *Jak2*^VF^ expression generated circulating mutated myeloid cells, leading to an increase in myocardial inflammation and HF through a signal transducer and activator of transcription (STAT)-1 phosphorylation-dependent mechanism [68]. Moreover, myeloid-restricted *Jak2*^VF^ mutation was also associated with increased expressions of IL-6 and IL-1β and an increased ability of macrophages to migrate to the heart.

**Figure 1:**
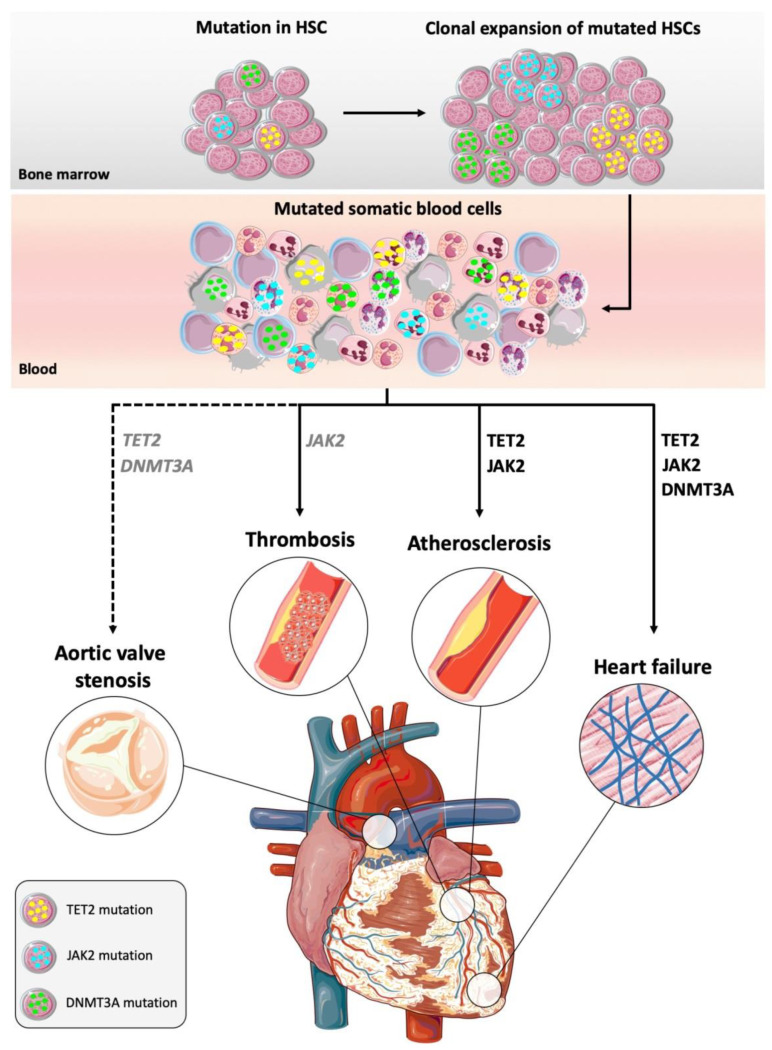
Clonal hematopoiesis of indeterminate potential (CHIP) causes cardiovascular disease. Mutations can confer a selective advantage to a hematopoietic stem cell (HSC) that leads to clonal expansion of the mutated HSC, which can give rise to a significant proportion of clones of mutated somatic blood cells. TET2 and JAK2 mutations preferentially involve myeloid cells. DNTM3A mutations are present in somatic blood cells of both myeloid and lymphoid origin. Mutations in the genes TET2, DNMT3A and JAK2 (in black) are associated with increased cardiovascular risk in epidemiological studies, and they have been causally implicated in cardiovascular diseases in animal models. TET2 and DNMT3A mutations (in grey italic) are enriched in aortic valve stenosis, but causality has not been established yet (indicated with dashed lines). JAK2 (in grey italic) hyperactivation is associated with thrombosis in CHIP patients; causality between JAK2 mutations and thrombotic diseases has been established in an animal model of myeloproliferative neoplasm (MPN).

Collectively, these findings indicate that JAK2 hyper-activation can promote CVD by altering hematopoiesis and then promoting thrombotic diseases. In addition, JAK2 mutations may favor CVD by boosting innate immunity responses, and this may explain how JAK2-driven CHIP favors atherosclerosis and HF (see Figure 1 for a comprehensive mechanistic view of CHIP and cardiovascular risk).

### 4.3. CHIP, The Chronic Inflammatory State and Ageing: One Culprit for Different Pathologies?

Evidence obtained in animal models shows that an increase in inflammation induced by CHIP-related mutations, worsens both atherosclerosis and HF [20,21]. Recent data in humans confirmed the inflammatory nature of atherosclerosis and the rationale of targeting inflammatory pathways as a valuable therapeutic tool [69]. However, even more recent data demonstrated that not all the strategies aimed to interfere with inflammatory pathways are effective in atherosclerosis [70]. The relationship between CHIP and chronic inflammation (and subsequent heightened vascular risk) is reported: CHIP causes an increase [71,72] in inflammatory responses and, at the same time, cells carrying CHIP mutations have a fitness advantage under pro-inflammatory conditions [73]. An interesting clinical translation of these findings is the recent observation that suggests a relationship between CHIP and HF [22,74]. Among prominent risk factors in HF genesis, age and systemic inflammation play a prominent role, and the most commonly mutated CHIP-driver genes (TET2 and DNMT3A) are associated with poor prognosis [22,31,39,74] in HF [22,74], even with different effects on the hematopoiesis driven by the two mutated genes. It is conceivable that a relationship exists between CHIP, the inflammatory state that worsens as we age [75], and the increased vascular risk in the elderly, with one factor influencing the other and vice-versa [75]. Furthermore, old age is usually associated with higher levels of IL-6, IL-1 receptor antagonist (IL-1ra), IL-18, C-reactive protein (CRP), fibrinogen, and soluble IL-6 receptor (sIL-6r) [75]. The latter study supports a tight correlation of inflammatory signals with CVD. However, we do not yet have a clear-cut biomarker of “inflammaging”, because not all the known inflammatory markers increase in older age [76]. Which comes first: cytokine or disease? The most recent data point towards a heightened immune activation and sustained inflammatory state, as an “adjuvant” of the vascular phenotype linked to the mature-age and elderly [76,77,78]. At the same time, CHIP could qualitatively influence the “inflammatory pabulum” and increase pro-inflammatory cytokine secretion [1,76,77]. A recent paper by Bick et al. [78] seems to support this scenario, because the attenuation of IL-6 signaling, caused by a genetic IL-6r variant, reduced cardiovascular risk in CHIP carriers. In addition, they reported a modest increase in hs-CRP (high sensitivity C-reactive protein) in the univariate analysis but not in the co-variate–adjusted analysis [78]. Moreover, data (not yet peer-reviewed) by the same group [79] claim CHIP association with IL-6 and IL-β levels, but not with hs-CRP (in a wide analysis of 32 cohorts, with 97,691 individuals and identifying 4229 CHIP cases). On the contrary, a very recent study by Busque et al. [80] shows that high-sensitivity (hs)-CRP is significantly higher in subjects with CHIP versus subjects without CHIP (analyzing, in total, 1887 subjects), supporting the hypothesis that a link between inflammation and CHIP-linked increased risk of CVD pathogenesis exists in ageing individuals. Despite these differences, taken together, these data confirm the association between inflammation, CHIP and aging. 

Likely, CHIP mutation load increases pro-inflammatory cytokine release, fueling a feedback loop in which several inflammatory molecules promote clonal expansions of mutated clones, which then amplify dysregulated and unbalanced pro-inflammatory cytokine release. Importantly, CHIP mutations involve myeloid cells, such as granulocytes, monocytes and NK cells, more frequently than B or T cells [81,82]. In mice with ischemic HF, *Tet2* loss-of-function mutations restricted to myeloid cells are sufficient to worsen cardiac function [47]. *Jak2^VF^* mutations in myeloid progenitors give rise to monocytes and neutrophils with increased inflammatory features, causing myocardial inflammation and accelerated HF after ischemic injury [68]. *Dntm3a* mutations result in a less pronounced bias towards myeloid cells [81,82]. However, mice transplanted with *Dnmt3a*-deficient HSCs, infused with angiotensin III to cause cardiac dysfunction, have displayed increased macrophages infiltration and increased transcription of markers of both innate and adaptive immune cells in the myocardium [48]. Data from animal models seem consistent with evidence from clinical trials showing that subjects with TET2 mutations have more inflammatory non-classical monocytes, while those with DNMT3A mutations have an increased an Th17/Tregs ratio [42]. Overall, these findings indicate that TET2 and JAK2 mutations preferentially increase inflammation mediated by innate immunity; otherwise, DNTM3A mutations affect both myeloid and/or lymphoid cells.

## 5. Potential Strategies for Targeting High-Risk CHIP Mutations 

Animal model studies have revealed some of the mechanisms by which CHIP mutations promote CVD, paving the way for the development of possible therapeutic options to reduce CVD risk in CHIP carriers. However, interventions aimed at reducing the risk associated with CHIP have not been attempted in clinical settings, and whether this may translate into practice in the future is still uncertain. In this section, we will discuss hypothetical approaches that could be examined as potential interventions to mitigate the pathogenic potential of mutated clones.

### 5.1. Immune Therapy 

CHIP mutations drive an increase in inflammatory responses; at the same time, cells carrying CHIP mutations have a fitness advantage under pro-inflammatory conditions [47,48,50]. Hence, targeting inflammation may mitigate the effects of CHIP, both by reducing clonal expansion and the CHIP-dependent inflammation. In murine models of CHIP driven by *Tet2,* NLRP3 inhibitor MCC950 reduces IL-1β and atherosclerosis [44], and decreases fibrosis and hypertrophy following myocardial infarction [47]. Cai and collaborators demonstrated that in *Tet2*-knockout mice (*Tet2*-KO), inflammatory stress results in an increase in IL-6, and that, in turn, IL-6 produced by *Tet2*-deficient HSPCs promotes clonal expansion. In these mice, inhibition of IL-6 downstream effectors SHP2-STAT3 reduced both IL-6 production and the clonal expansion of *Tet2*-KO HSPC [83]. Monoclonal antibodies against specific cytokines such as IL-1β (canakinumab) and IL-6 (tocilizumab and clazakizumab) are already used in clinical practice or being tested in clinical trials to treat diseases in which cytokines are abnormally secreted [69,84,85]. Similarly, targeting inflammatory pathways mediated by IL-1β and IL-6 may mitigate the deleterious effects of inflammation, possibly improving CVD outcomes in CHIP carriers.

### 5.2. JAK2 Inhibitors

Wolach and collaborators [66] demonstrated that, in a deep-vein-stenosis murine model, NET formation and thrombosis are blunted by inhibition of *Jak2* with ruxolitinib. In a very recent study in rabbits fed a high-fat diet, ruxolitinib reduced the atherosclerosis induced in aorta by balloon injury [86]. Furthermore, it has been shown that the selective *Jak2* inhibition with fedratinib reduces the formation of atherosclerotic plaque in *Apoe*-/-mice [87]. Interestingly, Edelmann and collaborators [88] found that in a mouse model of *Jak2^VF^*-positive chronic myeloproliferative neoplasia (CMN), *Jak2* promotes venous thrombosis by activating β1 and β2 integrins in leukocytes and, thus, inhibition of integrin activation could prevent pathologic thrombus formation. Collectively, these findings suggest that inhibition of JAK2 or downstream integrins may alleviate thrombotic CVD in CHIP patients carrying JAK2 mutations.

### 5.3. Vitamin C to Compensate for TET2 Function

In a mouse model of *Tet2*-dependent leukemia, treatment with vitamin C restored *Tet2* activity in HSPCs [89]. Similarly, in human leukemia cells, vitamin C suppresses proliferation and induces a TET2-dependent gene signature [89]. Vitamin C as an adjunct to decitabine therapy in AML patients can boost TET2 activity [90]. Furthermore, it has been reported that vitamin C treatment alone induced clinical remission in a case of AML with mutations in TET2 and Wilms tumor protein 1 (WT1), a transcription factor that recruits TET2 to DNA [91]. However, whether vitamin C might rescue TET2 activity in patients without AML is still unknown.

### 5.4. Glucose-Lowering Drugs

**Figure 2:**
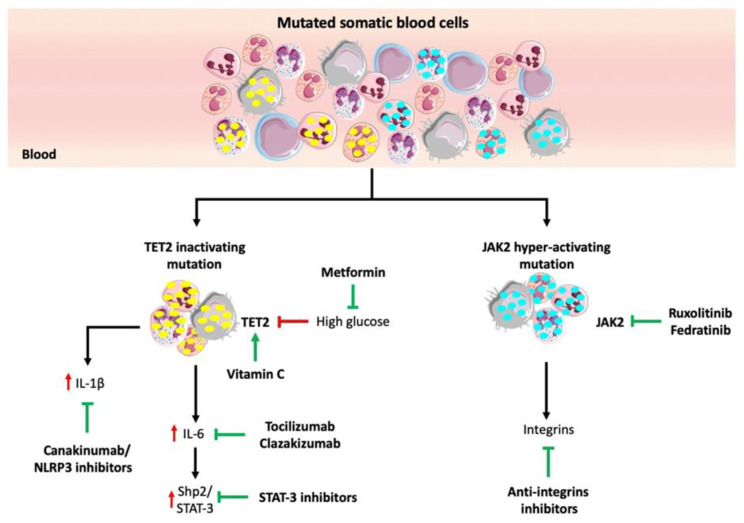
Potential strategies of targeting high-risk CHIP mutations. TET2 deficiency causes the increased expression of IL-1β and IL-6, which in turn activates the Shp2/STAT-3 signaling axis. Antibodies targeting IL-1β and IL-6, NLRP3 or STAT-3 inhibitors could mitigate the effect of a CHIP-mediated inflammation response. High glucose levels destabilize the TET2 protein. Lowering glucose by metformin may rescue TET2 activity. The mutagenic event that determines JAK2 hyperactivation is associated with thromboembolic events. JAK2 inhibitors (ruxolitinib or fedratinib) or anti-integrin inhibitors may reduce atherothrombotic complications.

CHIP carriers display a 30% increased risk of type 2 diabetes [14], which is a risk factor for both CVD and cancer [92]. However, it is not known yet whether CHIP promotes diabetes or whether the two conditions increase independently with age. In diabetic mice, high glucose destabilizes the TET2 protein, while lowering glucose with metformin rescues TET2 activity [93]. This finding suggests that high glucose may exacerbate the effect of TET2 mutations; on this basis, it could be hypothesized that a tight control of glucose levels may mitigate CVD risk in patients carrying TET2-driven CHIP. Figure 2 shows a synopsis of potential therapeutic strategies aimed to tackle CHIP-linked cardiovascular disease.

## 6. Conclusions and Future Perspectives

CHIP is a common, and perhaps inevitable, consequence of ageing. Individuals carrying these mutated clones are at increased risk, not only of developing hematological cancer, but also CVDs, such as atherosclerosis, stroke, thrombosis and HF. Our understanding of causative mechanisms linking CHIP to CVD is yet to be fully elucidated, and currently, no therapies are available that can reduce the risk of cancer or CVD for CHIP carriers.

The link between CVD and CHIP could be ascribed to inflammation, and it could be mediated by an inflammatory signaling pathway. A positive feedback mechanism of regulation and progression of the cross-talk between CHIP and CVD has been hypothesized: CHIP might induce inflammation and accelerate atherosclerosis, which in turn could further stimulate the expansion of mutated HSPC clones and their progeny, and further induce inflammatory responses and CVD progression. Clonal hematopoiesis may therefore contribute to the progression of CVD, by driving the release of several cytokines or augmenting the senescence of blood stem cells by altering the function of epigenetic modifiers. On the other hand, it should also be considered that the expansion of mutated HSC clones, characteristic of CHIP, may be boosted by classic cardiovascular risk factors, since hypercholesterolemia and diabetes could promote the expansion of HSPCs and myelopoiesis.

We have to point out that the mechanisms underlying the substantial association between CHIP and CVD are largely unknown at the moment, and the potential mechanisms illustrated above are hypothesis-generating. In this scenario, an intriguing possibility is that, as CHIP originates in the HSPC compartment, resulting from HSPC clones with DNA mutations conferring them a proliferation/survival-advantage (precancerous clonal expansion), similar mutations could influence senescence, apoptosis and/or differentiation, resulting in dysfunctional tissue-specific adult stem cells contributing to cardiovascular pathology and disease. Of note, the adult mammal heart harbors resident cardiac stem/progenitor cells (CSCs) that contribute to cardiovascular pathology with age [94,95,96]. Thus, CHIP could influence and modulate the processes of cardiovascular tissue repair/regeneration of the adult heart and vasculature by affecting the immune modulation of resident stem cells and yielding a pro-fibrotic inflammatory response over reparative/regenerative tissue re-shaping. This view widens our perspectives on the interplay between ageing, CHIP and CVD risk. In fact, ageing causes cellular and molecular changes in tissue homeostasis, and it is associated with telomere shortening as well as with a multi-component senescence-associated secretory phenotype (SASP), displaying pro-inflammatory cytokines, chemokines, extracellular matrix (ECM)-degrading proteins and other cell-cycle arrest signaling molecules [94,95,96,97]. All the above factors result in the progressive deterioration of the structure and function of any given organ at the tissue and cell levels [97]. CSC ageing leads to a senescent phenotype, and to a decreased ability to differentiate and to heal cardiac injuries. 

Trying to translate these new discoveries into clinical practice and the reality of public health systems worldwide, it is worth noting that in contemporary medicine, the development of new techniques for molecular diagnosis or advancements in other diagnostic fields leads to a constantly increasing proportion of patients who are not ill but at increased risk for specific illnesses. The number of abnormalities increases with age (as found in CHIP). We agree that, unless there are specific practical interventions that abrogate increased risk, the enhanced potential for diagnosis does not have an impact on clinically meaningful endpoints. Age is the most important cardiovascular risk factor, but the strategy to tailor therapy to the presence of CHIP is conceptual, yet still not formally proven at the moment. Whether CHIP would be an independent predictor of risk in a multivariable model with classical cardiovascular risk factors including age, is an open question. We can assume that there is potential for CHIP detection as a parameter for subject stratification where CVD risk is present, but we do not know at this stage whether knowledge of the presence of CHIP will improve therapy in CVD patients. 

In this regard, a recent publication by Sidlow and collaborators is trying to dissect and solve, at least in part, these uncertainties: it shows, in different case stories, the ways in which clinicians might face CHIP carriers and provide a suitable multidisciplinary protocol useful to address patients’ concerns in the clinical practice. This interesting and timely contribution shows three cases, ranging from casual recognition of CHIP to a diagnosis of TET2 mutation in a screening for evaluation of a stem cell transplant donor, to a patient treated for malignancy and in present good conditions with a somatic JAK^V621F^ mutation found in blood cells (for more details, see [20]).

The “take-home message” of this important contribution is that management of CHIP should be personalized and tailored with the age and life expectancy of the patient, concurring diseases and conditions and other CVD risk components. As witnessed by these cases, individuals with CHIP will increasingly ask for advice from CVD specialists regarding management of the condition, and a general suggestion can be extrapolated by this study. The challenge for practitioners is how to manage such individuals without a solid scientific and proven scaffold. Likely, increase in genome sequencing and increase in available data linking CHIP and CVD risk will increase the burden of guiding such patients on clinical practitioners. Further investigation into the clinical utility of CHIP to assess CVD risk is warranted, both in subjects who are apparently in good health status as well as in subjects with CVD or malignant disease.

In conclusion, the research in the area of CHIP is still new and evolving, and important hallmarks have been achieved that are beginning to clarify the role played by clonal hematopoiesis in the cardiovascular disease setting. DNMT3A, TET2 and also JAK2 mutations may promote an enhanced risk of CVD, but gene-specific differences could also exist and need to be fully elucidated in order to define the best targeted therapy in the cardiovascular disciplines. It is a rapidly evolving and promising field that nevertheless needs more experimental data and clinical studies before entering the armamentarium of cardiology and clinical medical practice.

## Figures and Tables

**Table 1:** Driver and passenger mutation, clone and clonality definitions [4,5].

Clone	Cells that share identical genomes within a cellular population.
Clonality	Historically refers to myeloid and lymphoid neoplasms; today denotes a uniform population of cells that could be either wild-type or mutant and eventually malignant.
Driver mutation	Driver mutations confer a growth advantage to the cells carrying them, and are positively selected during the evolution of cancer. They occur in a particular set of genes called “cancer genes”, and are often the primary causative agent of cancer.
Passenger mutation	Passenger mutations are inert somatic mutations. They are unable to confer clonal growth advantages, and therefore do not lead to cancer development. Nevertheless, passenger mutations are usually found in cancer cells’ genomes because when a driver mutation occurs, even a passenger mutation is carried together with the clonal expansion.

**Table 2:** Diagnostic criteria for clonal hematopoiesis of indeterminate potential (CHIP) [12,18].

Clonal hematopoiesis of indeterminate potential (CHIP)	Any clonal expansion of hematopoietic cells in a non-hematologic patient. It is characterized by - Absence of morphological variation in the blood cells, denoting morphological evidence of a hematological neoplasm; - Presence of a somatic mutation associated with hematological neoplasia, at a variant allele fraction of at least 2% in the peripheral blood; - Absence of diagnostic criteria for symptoms currently associated with hematological cancer (i.e., monoclonal gammopathy of undetermined significance or monoclonal B-cell lymphocytosis).

**Table 3:** List of studies investigating the link between CHIP and CVD in patients and animal models. BM, bone marrow; CHD, coronary heart disease; CVD, cardiovascular disease; HF, heart failure; HF/HC, high fat/high cholesterol; HSPC, hematopoietic stem/progenitor cells; LAD, left anterior descending artery; ldlr, low-density lipoprotein receptor; TAC, transverse aortic constriction; TAVI, transcatheter aortic valve implantation.

EPIDEMIOLOGICAL/ CLINICAL STUDIES			
STUDY/REFERENCE	C.H.I.P. STATUS	STUDY COHORT	MAIN FINDINGS
Jaiswal et al., 2014	Variants in 160 genes associated with hematological neoplasms.	17,182 subjects without hematological alterations.	CHIP is associated with increase in the risk of incident CHD and ischemic stroke. TET2, DNMT3A and ASXL1 mutations individually associated with CHD and ischemic stroke.
Jaywalk et al., 2017	Variants in 74 genes associated with hematological neoplasms.	4726 subjects with CHD and 3529 controls.	CHIP is associated with increased risk of CHD and early-onset MI. TET2, DNMT3, ASXL1 and JAK2 mutations individually associated with CHD and early-onset MI.
Dorsheimer et al., 2019	Variants in 56 genes associated with hematological neoplasms.	200 patients with HF following MI.	DNMT3A and TET2 carriers have increased death or HF re-hospitalization during a median follow-up of 4.4 years.
Mas-Peiro et al., 2019	Variants in TET2 and DNMT3A.	279 patients undergoing TAVI for severe aortic valve stenosis.	Patients with CHIP have increased all-cause mortality following successful TAVI during median follow-up of 9 months.
Bick et al., 2020	Variants in TET2 and DNMT3A.	35,416 subjects from UK Biobank without prevalent CVD.	CHIP is associated with increased risk of CVD. CHIP carriers with protective IL-6R variants have decreased CVD risk.
Abplanalp et al., 2020	Variants in TET2 and DNMT3A.	8 patients with severe aortic valve stenosis and 6 patients with HF.	CHIP carriers’ monocytes display a pro-inflammatory expression profile.
Wolach et al., 2018	JAK2VF variant	10,893 subjects without myeloid disorders.	JAK2VF mutations associated with increased risk of venous thrombosis.
**ANIMAL MODEL STUDIES**			
**STUDY**	**C.H.I.P. GENES**	**ANIMAL MODELS**	**MAIN FINDINGS**
Fuster et al., 2017	*Tet2*	Competitive BM transplant with *Tet2* -/- cells in irradiated *ldlr* -/- mice. HF/HC-induced atherosclerosis.	*Tet2* deficiency increases atherosclerotic plaque size and total number of macrophages in the intima of the vascular wall. *Tet2*-deficient macrophages show increase in NLRP3 inflammasome-mediated IL-1β secretion.
Jaiswal et al., 2017	*Tet2*	Irradiated *ldlr* -/- mice transplanted with *Tet2* -/+ or *Tet2* -/- BM cells. HF/HC-induced atherosclerosis.	*Tet2* deficiency increases atherosclerotic lesions in the aortic root and aorta. *Tet2*-deficient macrophages express more pro-inflammatory chemokines and cytokines.
Sano et al., 2018	*Tet2*	Competitive BM transplant with *Tet2* -/- cells in irradiated mice or conditional myeloid-restricted inactivation of *Tet2*. HF alternatively induced by TAC or LAD ligation.	*Tet2* deficiency worsens cardiac remodeling and function, and increases IL-1β expression.
Wang et al., 2020	*Tet2*	Non-preconditioned mice transplanted with *Tet2* -/+ or *Tet2* -/- BM cells.	*Tet2* deficiency causes age-related hypertrophy and fibrosis. Donor-derived macrophages in the heart have increased inflammatory features.
Sano et al., 2018	*Tet2 and Dnmt3a*	Irradiated mice transplanted with *Tet2* -/- or *Dnmt3a* -/- CRISPR-edited HSPCs. Angiotensin-II-induced HF.	*Tet2/Dnmt3a* mutations cause increased cardiac hypertrophy and fibrosis, and reduction in cardiac function.
Wang et al., 2018	*Jak2*	Irradiated *ldlr* -/- mice transplanted with Jak2^VF^-expressing BM cells. HF/HC-induced atherosclerosis.	*Jak2^VF^* mutation increases early and advanced atherosclerosis, promoting neutrophil infiltration and plaque instability. *Jak2^VF^* macrophages show increased pro-inflammatory cytokines and chemokines.
Sano et al., 2019	*Jak2*	Irradiated mice transplanted with HSPCs expressing *Jak2^VF^*. HF alternatively induced by TAC or LAD ligation.	*Jak2^VF^* mutation causes HF associated with increased expression of IL-6 and IL-1β.

CRISPR, clustered regularly interspaced short palindromic repeats.

## Abbreviations

CH	clonal hematopoiesis
CHIP	clonal hematopoiesis of indeterminate potential
CVD	cardiovascular disease
DNMT3A	DNA methyltransferase 3A
TET2	Tet methylcytosine dioxygenase 2
HSPC	hematopoietic stem/progenitor cells
MDS	myelo-dysplastic syndromes
ASXL1	additional sex combs-like transcriptional regulator 1
AML	acute myeloid leukemia
TP53	tumor protein p53
JAK2	janus kinase 2
SF3B1	splicing factor 3b subunit 1
CBL	casitas B-lineage lymphoma gene
SRSF2	serine and arginine rich splicing factor 2
PPM1D	protein phosphatase, Mg2+/Mn2+ Dependent 1D
BCOR	BCL6 Corepressor
HF	heart failure
NLRP3	NOD-, LRR- and pyrin domain-containing protein 3
CD163	Cluster Designation number 163, hemoglobin (Hb) scavenger receptor
CXCR4	C-X-C chemokine receptor type 4
CXCL12	C-X-C chemokine ligand 12
NOTCH1	Notch homolog 1, translocation-associated
MPN	myeloproliferative neoplasms
NET	neutrophil extracellular traps
CRP	C-reactive protein
SHP2	src homology region 2 (SH2)-containing protein tyrosine phosphatase 2
STAT3	signal transducer and activator of transcription 3
CMN	chronic myeloproliferative neoplasia
CSC	cardiac stem/progenitors cells
SASP	senescence-associated secretory phenotype
ECM	extracellular matrix
CRISPR	clustered regularly interspaced short palindromic repeats
hs-CRP	high sensitivity C-reactive protein
